# Inhibition of HIF-prolyl-4-hydroxylases prevents mitochondrial impairment and cell death in a model of neuronal oxytosis

**DOI:** 10.1038/cddis.2016.107

**Published:** 2016-05-05

**Authors:** S Neitemeier, A M Dolga, B Honrath, S S Karuppagounder, I Alim, R R Ratan, C Culmsee

**Affiliations:** 1 Institut für Pharmakologie und Klinische Pharmazie, Biochemisch-Pharmakologisches Centrum Marburg, Fachbereich Pharmazie, Philipps-Universität Marburg, Karl-von-Frisch-Straße 1, Marburg 35032, Germany; 2 Burke-Cornell Medical Research Institute, White Plains, NY, USA; 3 Feil Family Brain and Mind Research Institute, Department of Neurology and Neuroscience, Weill Medical College, Cornell University, New York, NY, USA

## Abstract

Mitochondrial impairment induced by oxidative stress is a main characteristic of intrinsic cell death pathways in neurons underlying the pathology of neurodegenerative diseases. Therefore, protection of mitochondrial integrity and function is emerging as a promising strategy to prevent neuronal damage. Here, we show that pharmacological inhibition of hypoxia-inducible factor prolyl-4-hydroxylases (HIF-PHDs) by adaptaquin inhibits lipid peroxidation and fully maintains mitochondrial function as indicated by restored mitochondrial membrane potential and ATP production, reduced formation of mitochondrial reactive oxygen species (ROS) and preserved mitochondrial respiration, thereby protecting neuronal HT-22 cells in a model of glutamate-induced oxytosis. Selective reduction of PHD1 protein using CRISPR/Cas9 technology also reduced both lipid peroxidation and mitochondrial impairment, and attenuated glutamate toxicity in the HT-22 cells. Regulation of activating transcription factor 4 (ATF4) expression levels and related target genes may mediate these beneficial effects. Overall, these results expose HIF-PHDs as promising targets to protect mitochondria and, thereby, neurons from oxidative cell death.

Neurodegenerative diseases such as Alzheimer's disease (AD), Parkinson's disease (PD) and stroke affect millions of people in the ageing societies worldwide and, thus, are of great clinical importance and scientific interest. Although they widely differ in pathology and symptoms, neurodegenerative diseases share common regulated pathways of neuronal cell death underlying manifestation and progression of these diseases. For example, enhanced oxidative stress has been established as a common characteristic and key mediator of neuronal demise.^1, 2, 3^ High amounts of reactive oxygen species (ROS) caused by glutamate overload, toxic intracellular Ca^2+^ concentrations or activation of lipoxygenases (LOX)^4, 5^ induce oxidative damage of proteins and lipids at the plasma membrane and in organelles, respectively, thereby leading to regulated cell death.^6^ Mitochondria, in particular, have a pivotal role in this cell death paradigm because they are the key organelles in the energy metabolism and regulation sites of ROS and apoptosis signaling pathways. Mitochondria are major targets of ROS as their membranes and DNAs are easily accessible and accordingly highly vulnerable to oxidative stress.^7^ They also significantly contribute to the additional formation of ROS when their own redox balance is impaired.^8^ Upon damage, mitochondria release proapoptotic proteins such as cytochrome *c* (Cytc), apoptosis-inducing factor (AIF) and endonuclease G,^9, 10^ which leads to cell death. Hence, it is well accepted that mitochondrial damage marks the so-called ‘point of no return',^10^ meaning that cells with impaired mitochondria cannot survive. Therefore, protection of mitochondria is a promising strategy against neuronal dysfunction and damage and, therefore, against the manifestation and progression of neurodegenerative diseases.

Recently, hypoxia-inducible factor (HIF) prolyl-4-hydroxylases (PHDs) emerged as promising target candidates for mitochondrial protection in paradigms of oxidative stress. The inhibition of HIF-PHDs prevented neuronal cell death induced by mitochondrial toxins.^11^ In PHD1^−/−^ myofibers mitochondrial respiration in response to hypoxia was preserved owing to reduced oxidative stress.^12^

HIF-PHDs belong to a family of dioxygenases depending on oxygen, iron and 2-oxoglutarate. They exist in the three isoforms, PHD1, PHD2 and PHD3, and act as oxygen sensors because of their main function being the regulation of HIF expression levels.^13^ However, more and more HIF-independent functions of HIF-PHDs^11, 14^ and alternative substrates^15, 16, 17^ have been recently identified, which are partly isoform specific. Genetic approaches revealed reduced infarct volumes in PHD1^−/−^ mice, and exposed improved behavior and less neuronal cell death in the penumbra in PHD2^+/−^ mice in a model of transient focal cerebral ischemia.^18^ Further, neuron-specific knockout of PHD2 confirmed neuroprotective effects in the CA1 region after transient cerebral ischemia in mice.^19^ Pharmacological inhibition of HIF-PHDs by iron chelators or 2-oxoglutarate analogs also provided neuroprotection *in vitro* and *in vivo*, in models of ischemic neuronal death,^20, 21, 22^ AD,^11, 23^ PD^24, 25, 26^ and Huntington's disease,^11^ further supporting the pivotal role of HIF-PHDs in neurodegeneration.

To pursue this promising strategy, new HIF-PHD inhibitors have been developed that are more selective than previously available iron chelators such as deferoxamine (DFO)^11, 14, 20^ and ciclopirox (CPO).^11^ In particular, branched oxyquinolines were identified to inhibit HIF-PHDs via coordination of iron at the active site of HIF-PHDs and modeling shows little predicted interaction with other 2-oxoglutarate-dependent dioxygenases. Additionally, branched oxyquinolines exhibit less iron-chelating properties than CPO,^27^ making it a useful tool to study the effects of selective HIF-PHD inhibition. We named this branched oxyquinoline molecule adaptaquin (AQ) for its ability to inhibit the HIF-PHDs and activate *adaptive* responses to hypoxia.^27^

In the present study, we investigated the effects of AQ-mediated HIF-PHD inhibition and PHD1 gene silencing on cell viability and, especially, mitochondrial integrity and function in a model of neuronal oxytosis to elucidate the mechanisms leading to AQ-mediated neuronal protection. Oxytosis is defined as oxidative cell death in response to glutamate toxicity, which induces a depletion of glutathione (GSH) and subsequent formation of ROS, resulting in mitochondrial demise and cell death. We found comparable effects of AQ and PHD1 gene silencing on both mitochondrial function and cell viability, suggesting a crucial role for HIF-PHDs in mitochondrial impairment and subsequent neurodegeneration induced by oxidative stress.

## Results

### PHD1 gene silencing attenuates oxytosis and restores mitochondrial function

**Figure 1:**
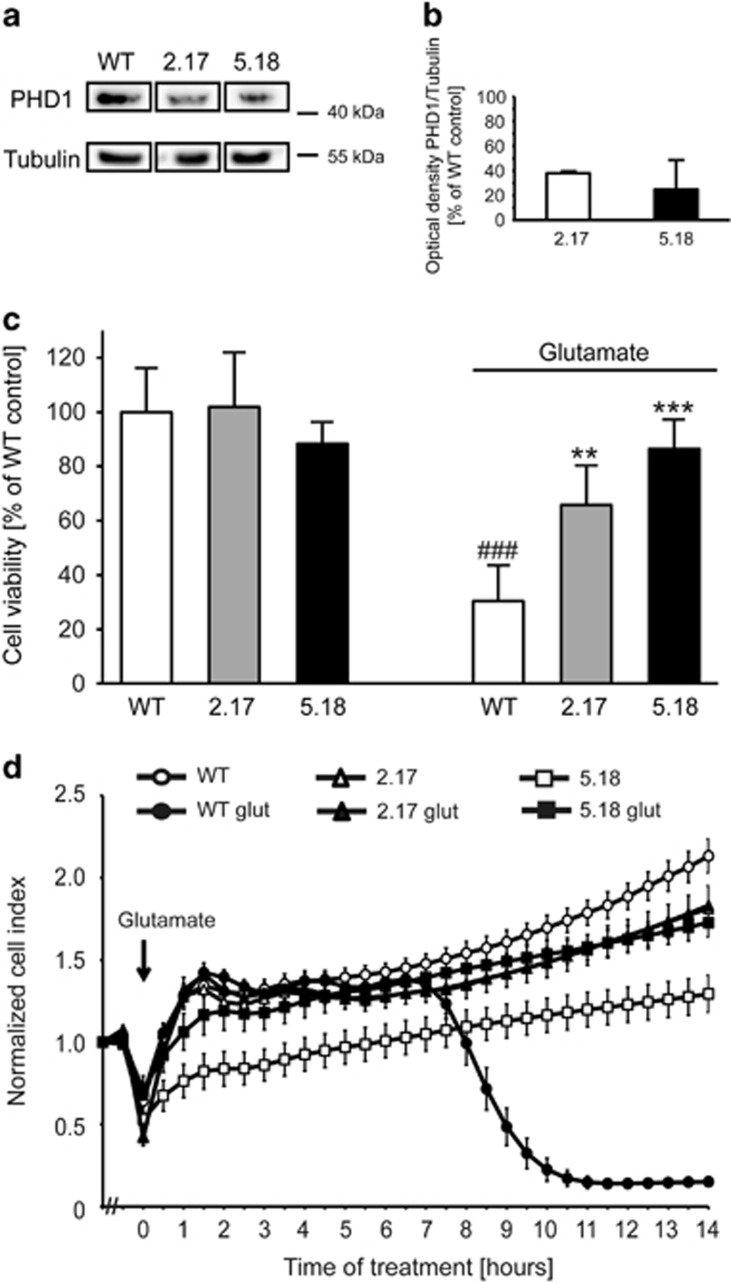
Silencing PHD1 by CRISPR/Cas9 attenuates oxytosis. (**a**) Selective knockdown of PHD1 by CRISPR/Cas9 was verified by western blot analysis. (**b**) Quantification of four independent western blots shows a reduction of PHD1 protein level. (**c**) MTT assay reveals protection of the downregulation of PHD1 by CRISPR/Cas9 (clones 2.17 and 5.18) against glutamate toxicity (5 mM, 16 h) compared with WT cells. Data are given as mean±S.D. (*n*=8). ^###^*P*<0.001 compared with untreated WT control; **P<0.01 and ***P<0.001 compared with glutamate-treated WT control (ANOVA, Scheffé's test). (**d**) xCELLigence real-time measurement: downregulation of PHD1 by CRISPR/Cas9 (clones 2.17 and 5.18) shows protection against treatment with 7 mM glutamate (glut) compared with WT cells

In HT-22 cells, high concentrations of extracellular glutamate induce lethal oxidative stress.^28^ This cell death paradigm is known as oxytosis and is characterized by significant morphological and metabolic changes, for example, GSH depletion or activation of LOX.^29^ To study the role of PHD1 in this model system of lethal oxidative stress, we used CRISPR/Cas9 technology to create cells with reduced expression of PHD1 protein. From the initial screening two clones were selected, referred to as clone 2.17 and clone 5.18 below. Western blot analysis (Figure 1a) and subsequent quantification (Figure 1b) revealed significant downregulation of PHD1 protein in both clones compared with wild-type (WT) HT-22 cells. We further analyzed the effects of this downregulation of PHD1 on cell viability and mitochondrial function. PHD1 silencing attenuated glutamate-induced loss of cell viability indicated by MTT (3-(4,5-dimethylthiazol-2-yl)-2,5-diphenyltetrazolium bromide) assay (Figure 1c). This protective effect was permanent at least for 14 h of the recording, as further detected by real-time measurements of cellular impedance (Figure 1d).

**Figure 2:**
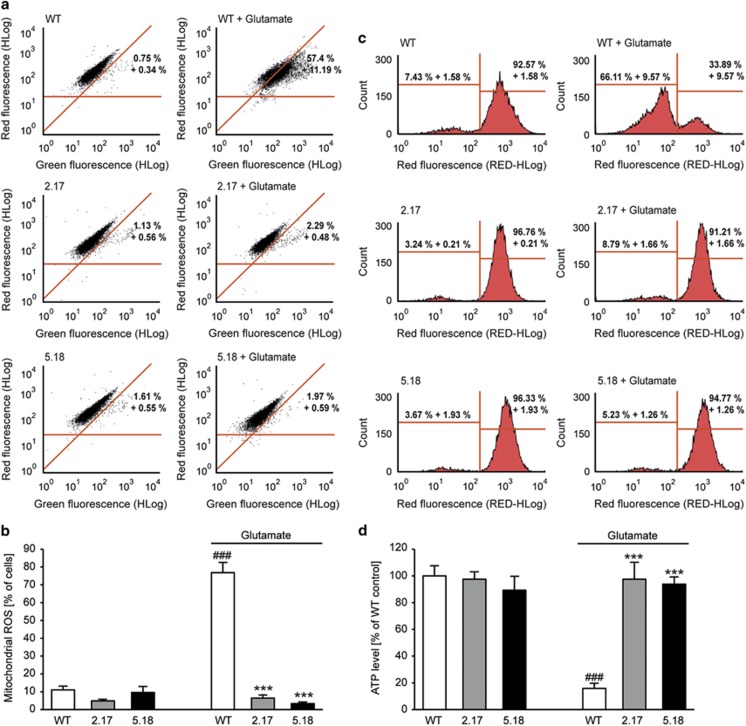
PHD1 silencing by CRISPR/Cas9 restores mitochondrial function and prevents ROS formation. (**a**) Cells were stained with BODIPY 581/591, a lipophilic fluorescent dye that undergoes a shift from red to green fluorescent emission upon oxidation by free radicals and therefore correlates with the formation of lipid peroxides. Changes of fluorescence were detected by FACS analysis after 15 h of glutamate treatment (7 mM). Clones 2.17 and 5.18 showed a significant reduction of the lipid peroxide production compared with WT control cells (*n*=4). (**b**) Mitochondrial ROS production was detected by MitoSOX staining and following FACS analysis. Glutamate treatment (7 mM, 15.5 h) led to an increase in ROS production. Downregulation of PHD1 by CRISPR/Cas9 (clones 2.17 and 5.18) reduced this increase. Quantification of MitoSOX fluorescence of *n*=4 independent experiments. Data are shown as mean±S.D. ^###^*P*<0.001 compared with untreated WT control; ***P<0.001 compared with glutamate-treated WT control (ANOVA, Scheffé's test). (**c**) Representative FACS plots showing that MMP measured by TMRE fluorescence is restored by downregulation of PHD1 by CRISPR/Cas9 (clones 2.17 and 5.18) after glutamate exposure (6 mM, 15.5 h). (**d**) After 15.5 h of treatment with glutamate (7 mM), ATP levels were measured. PHD1 silencing by CRISPR/Cas9 (clones 2.17 and 5.18) prevented glutamate-induced ATP depletion (*n*=8). Data are shown as mean±S.D. ^###^*P*<0.001 compared with untreated WT control; ***P<0.001 compared with glutamate-treated WT control. (ANOVA, Scheffé's test)

In the model system of oxytosis, enhanced lipid peroxidation is regarded as a trigger for mitochondrial damage.^30^ Hence, we examined the formation of lipid peroxides after PHD1 gene silencing in the presence and absence of glutamate. BODIPY staining and consequent fluorescence-activated cell sorting (FACS) analysis revealed that PHD1 downregulation prevented lipid peroxidation upon glutamate treatment and did not affect basal levels of lipid ROS (Figure 2a). As it is well established that mitochondrial demise due to elevated ROS levels is a hallmark of oxytosis,^30, 31^ mitochondrial ROS formation was then analyzed. To this end, WT HT-22 cells, clone 2.17 and clone 5.18 were treated with glutamate and stained with MitoSOX. Subsequent FACS analysis showed an increase in mitochondrial superoxide formation, which was significantly abolished by PHD1 downregulation (Figure 2b). As previously described in this system of glutamate toxicity in HT-22 cells,^32, 33^ the oxidative cell death is associated with a breakdown of mitochondrial membrane potential (MMP). Measurement of MMP revealed a reduction of mitochondrial depolarization by selective PHD1 silencing indicated by restored tetramethylrhodamine ethyl ester (TMRE) fluorescence (Figure 2c). These findings correlated well with the corresponding effects on cell viability. Further, we observed a pronounced loss of ATP after onset of glutamate, which was fully abolished by PHD1 gene silencing (Figure 2d).

To exclude off-target effects, we used siRNA approaches to transiently knockdown PHD1 and performed the same set of experiments. PHD1 siRNA sequences I and II (siPHD I and siPHD II) significantly decreased the mRNA levels of PHD1, while the downregulation at the protein level was not as pronounced (as shown in the western blot in Supplementary Figure 1a). The effect of the siRNAs on PHD1 protein was smaller than the one achieved by the CRISPR/Cas9 technology. Quantification of western blots of five independent experiments confirmed a regulatory effect of the siRNA sequences targeting PHD1 at the protein level, and also showed a high variation of gene silencing efficacy (Supplementary Figure 1b). This partial siRNA-mediated PHD1 downregulation attenuated glutamate-induced loss of cell viability indicated by MTT assay (Supplementary Figures 1c and d). Opposed to the findings in clone 2.17 and 5.18, but in line with the partial downregulation of the PHD1 protein, this protective effect was only transient when detected by real-time measurement of cellular impedance (Supplementary Figures 1e and f). However, knockdown of PHD1 by siRNA approaches attenuated all hallmarks of glutamate-induced oxidative cell death in HT-22 cells, such as the formation of mitochondrial and lipid peroxides, the breakdown of MMP and loss of ATP (Supplementary Figure 2).

### AQ protects against glutamate-induced cell death

**Figure 3:**
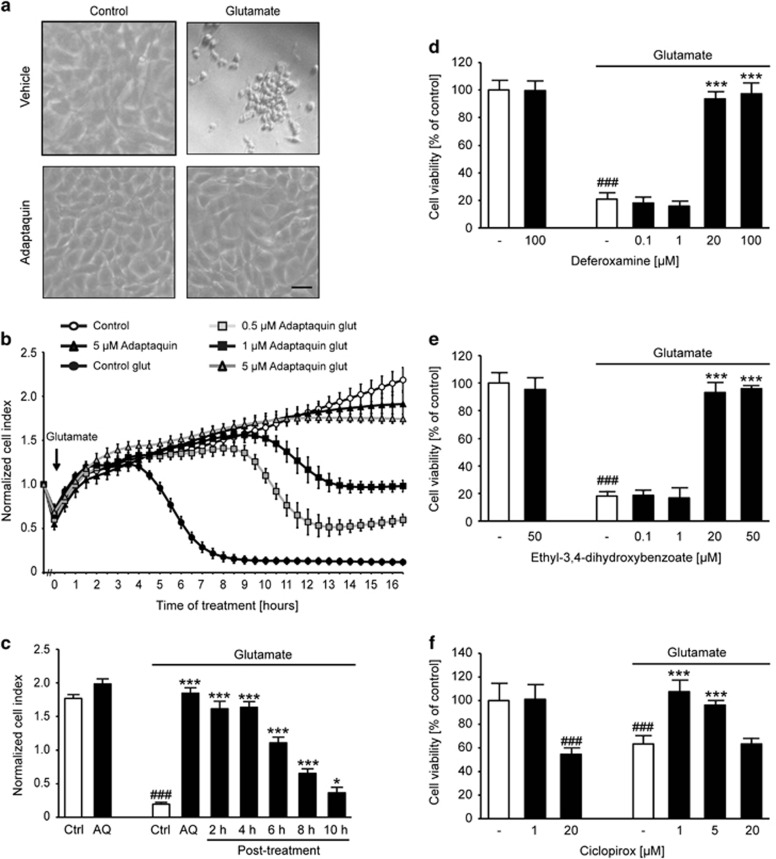
PHD inhibitors protect against oxytosis. (**a**) Light microscopy pictures show altered morphology of glutamate-treated (5 mM, 15 h) HT-22 cells. Cotreatment with AQ (2 *μ*M) fully prevents these changes. Scale bar: 50 *μ*m. (**b**) xCELLigence real-time impedance measurement shows dose-dependent salutary effect of AQ (*n*=6). (**c**) Bar graph evaluation at the 15.5 h time point from xCELLigence recordings (Supplementary Figure 3b, right black arrow) under post-treatment conditions (*n*=6). ^###^*P*<0.001 compared with untreated control; *P<0.05 and ***P<0.001 compared with glutamate-treated control (ANOVA Scheffé's test). (**d–f**) MTT assays show protective effects of different concentrations of DFO (**d**), DHB (**e**) and CPO (**f**) against glutamate-induced (7 mM, 16 h) oxytosis (*n*=8). Data are given as mean±S.D. ^###^*P*<0.001 compared with untreated control; ***P<0.001 compared with glutamate-treated control (ANOVA Scheffé's test)

To further investigate the effects of HIF-PHD inhibition during oxytosis, we used the pharmacological selective HIF-PHD-inhibitor AQ.^27^ The glutamate challenge induced established morphological hallmarks of cell death, that is, the cells shrink, round up and detach from the bottom of the culture dish. Cotreatment with AQ fully prevented these morphological features of cell death (Figure 3a) and preserved cell viability as detected by the MTT assay (Supplementary Figure 3a). A dose-dependent protective effect of AQ was further detected using the xCELLigence system (Roche, Penzberg, Germany; Figure 3b). To establish a therapeutic time window for AQ in the paradigm of glutamate-induced oxytosis, we treated HT-22 cells with the HIF-PHD inhibitor 2 at 4, 6, 8 and 10 h following the onset of glutamate. Real-time recordings of the cellular impedance demonstrated full protection against oxytosis by post-treatment with AQ up to 4 h after the onset of glutamate exposure (Supplementary Figure 3b). Notably, even 10 h after the onset of glutamate, AQ was able to rescue a significant part of the cells (Figure 3c), indicating a very strong neuroprotective potential of this particular HIF-PHD inhibitor with an extended post-treatment window of up to 10 h after onset of oxidative stress.

Glutamate-induced calcium influx is a late-stage event during oxytosis.^34^ Under conditions of extracellular calcium depletion, AQ was still able to prevent glutamate-induced cell death (Supplementary Figure 3c). This observation indicates that AQ does not act via a chelation of calcium and underlines its target specificity.

To confirm the protective effect of HIF-PHD inhibition in the model system of neuronal oxytosis, we next applied different inhibitors of HIF-PHDs, including the 2-oxoglutarate analog ethyl-3,4-dihydroxybenzoate (DHB: 0.1–50 *μ*M) as well as the iron chelators DFO (0.1–100 *μ*M) and CPO (1–20 *μ*M). All HIF-PHD inhibitors tested protected HT-22 cells against glutamate-induced oxytosis in a similar manner as AQ, which was indicated by preserved cell viability (Figures 3d–f).

### AQ preserves mitochondrial respiration

**Figure 4:**
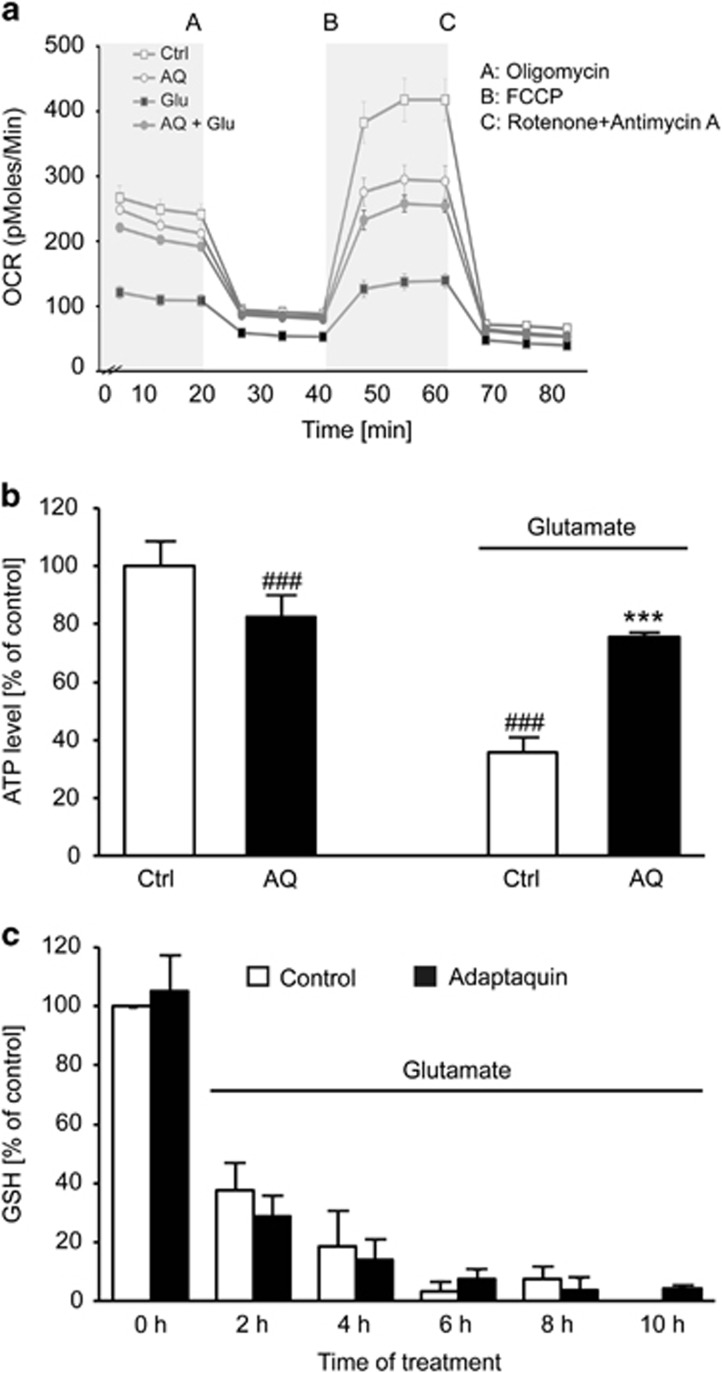
AQ preserves mitochondrial respiration. (**a**) Measurement of the OCR reveals restored basal and maximal respiration by AQ (2 *μ*M) after glutamate exposure (4 mM, 15 h). (**b**) Quantification of ATP production from (**a**) calculated as difference between average OCR in section A (light gray) and section B (white) shows loss of ATP after glutamate exposure. AQ prevents decrease of ATP levels (*n*=6). Data are given as mean±S.D. ^###^*P*<0.001 compared with untreated control; ***P<0.001 compared with glutamate-treated control (ANOVA Scheffé's test). (**c**) Measurement of GSH depicts rapid decrease of GSH after glutamate exposure (5 mM), which is not restored by cotreatment with AQ (2 *μ*M)

Mitochondria are known to have a key role in cellular life and death, because they are the main source of energy supply, regulation sites for ROS production and key control points for intrinsic pathways of apoptosis.^35^ Impaired energy metabolism due to mitochondrial damage is reflected by reduced ATP levels and loss of MMP, which are regarded as hallmarks of neuronal cell death.^1, 10^ To correlate the observed neuroprotective properties of AQ with restored mitochondrial function, we examined the mitochondrial respiratory capacity and ATP production by measuring the oxygen consumption rate (OCR) using the Seahorse XF96 system (Seahorse Biosciences, North Billerica, MA, USA). The glutamate challenge decreased both the basal and maximal respiration reflected by a reduced OCR. These detrimental metabolic changes were abolished by cotreatment with AQ (Figure 4a), which, furthermore, restored the mitochondrial ATP production (Figure 4b).

Owing to the very pronounced effect of AQ on cell viability, as well as mitochondrial integrity, we were interested to find out if this particular HIF-PHD inhibitor was capable of preventing glutamate-induced GSH depletion, which is the starting point of oxidative stress and oxytosis. To this end, we examined the levels of GSH after the glutamate challenge in the presence and absence of AQ. Similar to previous findings,^29, 34^ glutamate (5 mM) induced a rapid and sustained decrease of GSH levels within 2 h and up to 10 h of exposure. Notably, we were not able to detect GSH levels in HT-22 cells after 10 h of glutamate exposure because the GSH levels dropped below detection limits. Surprisingly, although AQ fully protected HT-22 cells from cell death and morphological changes, it failed to rescue the observed drop of GSH levels (Figure 4c). These findings indicated that HIF-PHD inhibition provided neuroprotective effects downstream of GSH depletion, and GSH restauration was dispensable for the protective effects.

**Figure 5:**
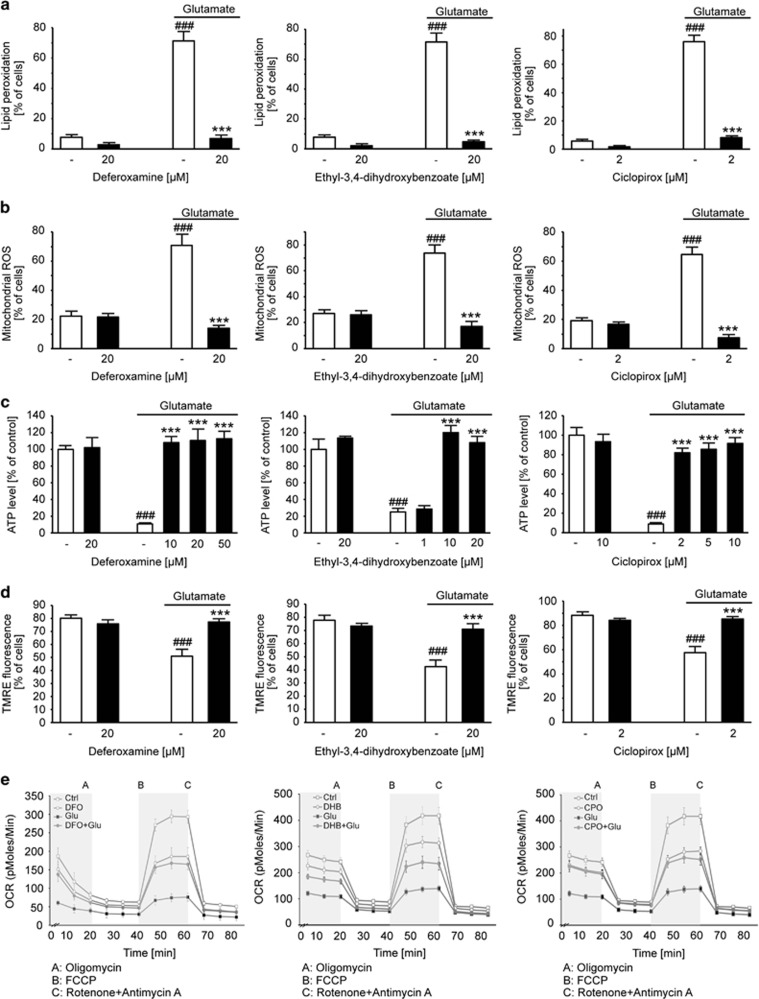
PHD inhibitors preserve mitochondrial function and prevent generation of mitochondrial and lipid peroxides. (**a**) DFO (20 *μ*M), DHB (20 *μ*M) and CPO (2 *μ*M) abolish glutamate-induced (7 mM, 15 h) formation of lipid peroxides measured by BODIPY staining and subsequent FACS analysis. Data are given as mean±S.D. (*n*=4). ^###^*P*<0.001 compared with untreated control; ***P<0.001 compared with glutamate-treated control (ANOVA Scheffé's test). (**b**) DFO (20 *μ*M), DHB (20 *μ*M) and CPO (2 *μ*M) prevent generation of mitochondrial ROS upon glutamate treatment (7 mM, 15 h). Data are given as mean±S.D. ^###^*P*<0.001 compared with untreated control; ***P<0.001 compared with glutamate-treated control (ANOVA Scheffé's test). (**c**) Different concentrations of DFO, DHB and CPO prevent glutamate-induced (7 mM, 15 h) loss of ATP. Data are given as mean±S.D. (*n*=8). ^###^*P*<0.001 compared to untreated control; ***P<0.001 compared to glutamate-treated control; ANOVA Scheffé's test. (**d**) DFO (20 *μ*M), DHB (20 *μ*M) and CPO (2 *μ*M) fully prevent breakdown of MMP after glutamate challenge (7 mM, 15 h). Data are given as mean±S.D. (*n*=4). ^###^*P*<0.001 compared with untreated control; ***P<0.001 compared with glutamate-treated control; ANOVA Scheffé's test. (**e**) Measurement of the OCR after glutamate exposure (4 mM, 15 h) shows decrease of the basal and maximal respiration, which was prohibited by DFO (20 *μ*M), DHB (20 *μ*M) and CPO (2 *μ*M), respectively

The other aforementioned HIF-PHD inhibitors exhibited similar effects upstream and at the level of mitochondria as observed for AQ. DFO, DHB and CPO prevented glutamate-induced lipid peroxidation (Figure 5a) and subsequent formation of mitochondrial ROS (Figure 5b), while none of the inhibitors affected any of these parameters under basal conditions. Furthermore, DFO, DHB and CPO fully restored ATP levels (Figure 5c) and the MMP examined via TMRE staining (Figure 5d). Similar to the effects obtained with AQ, DFO, DHB and CPO also maintained both, basal and maximal mitochondrial respiration (Figure 5e). These observations suggest an almost intact mitochondrial function upon inhibition of HIF-PHDs in the presence as well as in the absence of glutamate, which correlates well with the observed protection of cell viability.

### AQ shows antioxidative properties independent of MnSOD

**Figure 6:**
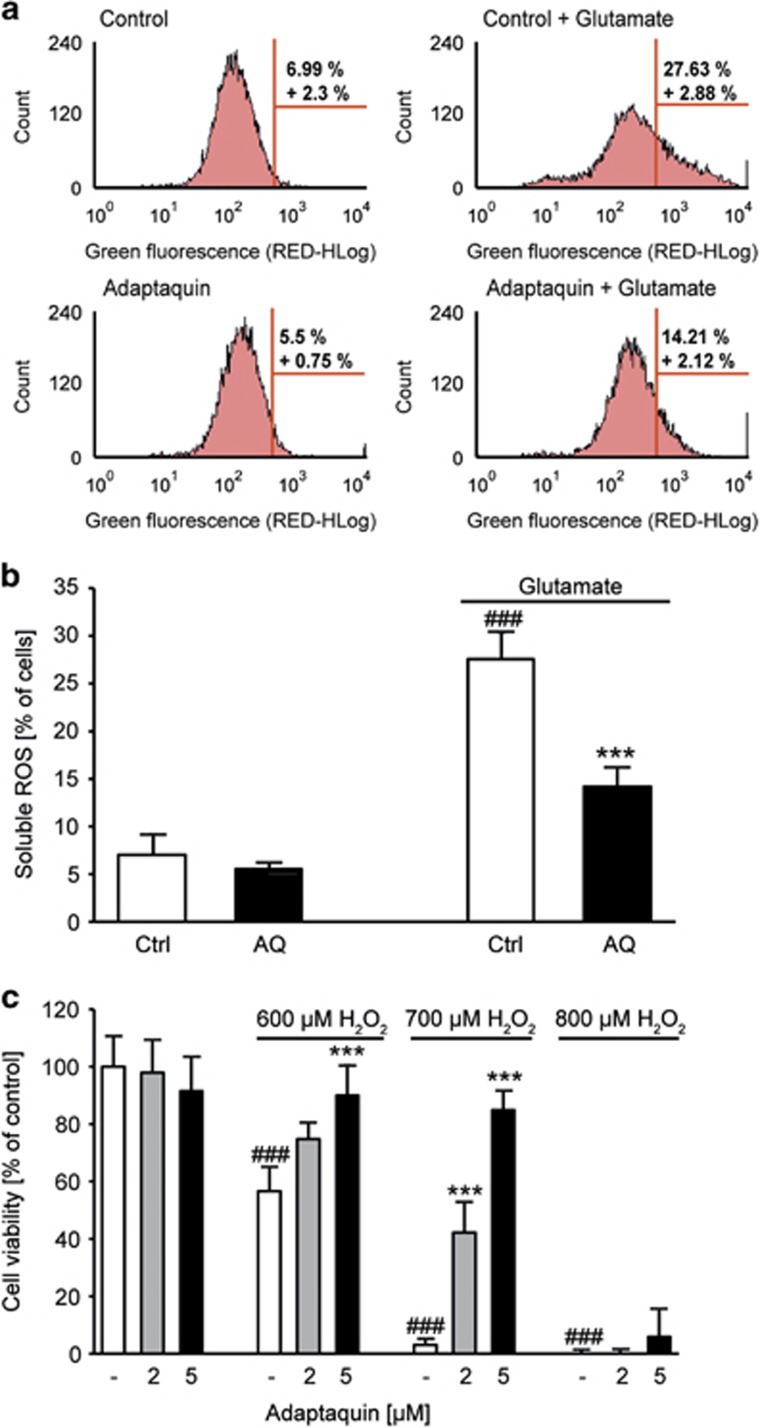
AQ shows small antioxidative properties. (**a**) DCF staining shows formation of soluble ROS after 6 h of glutamate (4 mM) challenge. Cotreatment with AQ (2 *μ*M) reduces soluble ROS. (**b**) Quantification of DCF fluorescence of *n*=3 independent experiments shown in the representative plots in (**a**). Data are shown as mean±S.D. ^###^*P*<0.001 compared with untreated control; ***P<0.001 compared with glutamate-treated control; ANOVA, Scheffé‘s test. (**c**) MTT assay shows dose-dependent protection of AQ against H_2_O_2_-induced cell death (16 h). Data are shown as mean±S.D. (*n*=8). ^###^*P*<0.001 compared with untreated control; ***P<0.001 compared with respective H_2_O_2_-treated control; (ANOVA, Scheffé's tes)

As GSH is the main antioxidant in the brain, it has a crucial role for detoxification of ROS and a disturbed GSH metabolism has been associated with neurodegenerative diseases.^36^ The question arose of how can AQ mediate neuroprotection despite impaired GSH levels. To address this question, we analyzed the formation of soluble ROS 6 h after the onset of glutamate treatment to identify possible antioxidative characteristics of AQ. To this end, HT-22 cells were stained with CM-H_2_DCFDA (5,6-chloromethyl-2′,7′-dichlorodihydrofluorescein diacetate acetyl ester), a fluorescent dye that shows green fluorescence upon oxidation by soluble ROS. After 6 h of glutamate exposure, we saw an increase in green fluorescence, indicating enhanced formation of ROS (Figure 6a), which correlates well with the results of the GSH measurements. Hence, AQ reduced this effect, but was not able to fully abolish it (Figure 6b). This suggests some antioxidative properties, which were further supported by a dose-dependent protection against H_2_O_2_-induced cell death (Figure 6c). However, these partial antioxidative effects cannot fully explain the mitochondrial protection, and, therefore, we also checked for changes in the expression of the manganese superoxide dismutase (MnSOD). MnSOD is known to be part of the antioxidative defense of mitochondria and its overexpression has been shown to reduce oxidative damage in HT-22 cells.^28^ Interestingly, glutamate induced a strong increase in MnSOD levels while they remained almost unchanged after treatment with AQ (Supplementary Figure 4a). Thus, suggesting that AQ does not mediate protection against glutamate by enhancing the antioxidative defense machinery of mitochondria. The glutamate-induced upregulation of MnSOD indicates a compensatory effect of the damaged cells to cope with enhanced oxidative stress.^37^

### AQ alters ATF4 expression

**Figure 7:**
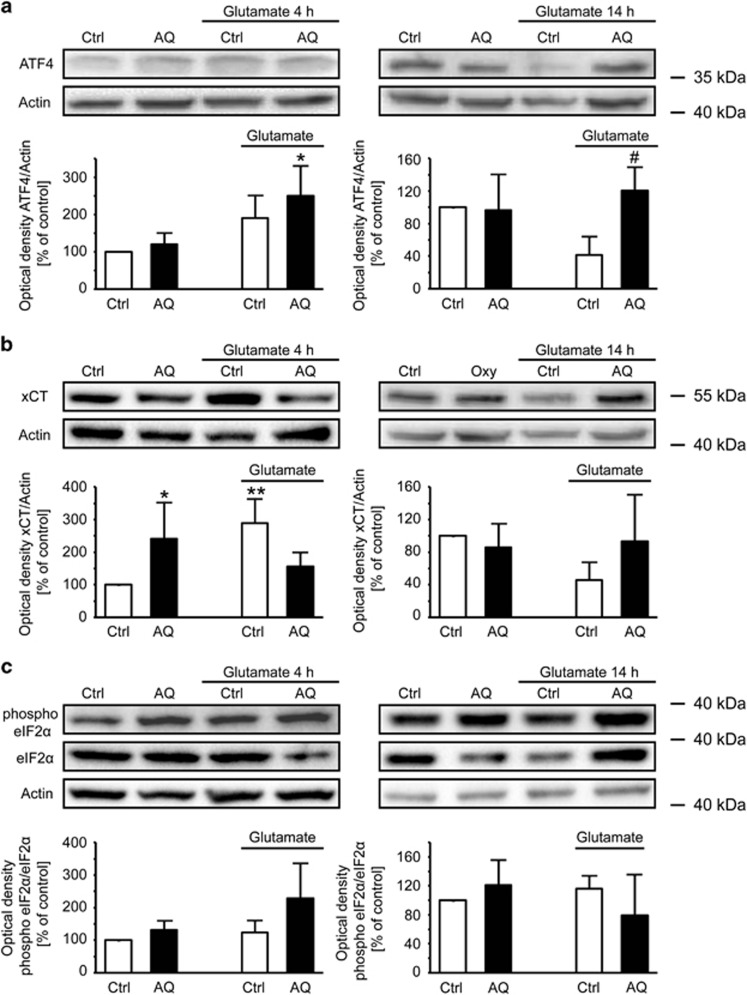
AQ regulates ATF4 expression. (**a**) Representative western blots and corresponding quantifications of four independent experiments show upregulation of ATF4 after 4 h of glutamate and downregulation after 14 h, which is restored by AQ. ^#^*P*<0.05 compared with glutamate-treated control. (**b**) Representative western blots and corresponding quantifications of four independent experiments reveal upregulation of xCT after 4 h of glutamate and downregulation after 14 h, which is restored by AQ. *P<0.05 and **P<0.01 compared with untreated control. (**c**) Representative western blots and corresponding quantification do not indicate significant changes of the phosphorylation state of eIF2*α* upon glutamate challenge or treatment with AQ

As ATF4 has been shown to correlate with oxidative stress^38, 39^ and also to regulate and interact with HIF-PHDs,^40, 41^ we examined the expression of ATF4 upon glutamate exposure to further elucidate the mechanism of AQ-mediated protection. ATF4 expression increased 4 h after the onset of glutamate treatment and decreased at 14 h. This downregulation associated with glutamate-induced death was fully prevented by HIF-PHD inhibition (Figure 7a). A similar expression pattern after 14 h of glutamate exposure was found for the xCT, which was recently shown to be regulated by ATF4 in HT-22 cells.^42^ After 14 h of glutamate treatment, xCT levels declined, while cotreatment with AQ restored xCT expression levels at this time point (Figure 7b). To determine if the regulation of xCT was driven via the eukaryotic initiation factor 2*α* (eIF2*α*)/ATF4 pathway, we investigated the phosphorylation state of eIF2*α* at both 4 and 14 h after the onset of the glutamate treatment. The phosphorylation of eIF2*α* seemed to be transiently enhanced at 4 h in the presence of AQ irrespective of glutamate exposure. After 14 h, none of the treatment conditions induced significant changes in the phosphorylation state of eIF2*α* (Figure 7c). Overall, these data imply that the alterations in xCT expression levels may be mediated through ATF4, but are not induced via the eIF2*α*/ATF4 pathway.

### ATF4 expression is not required for AQ-mediated protection

**Figure 8:**
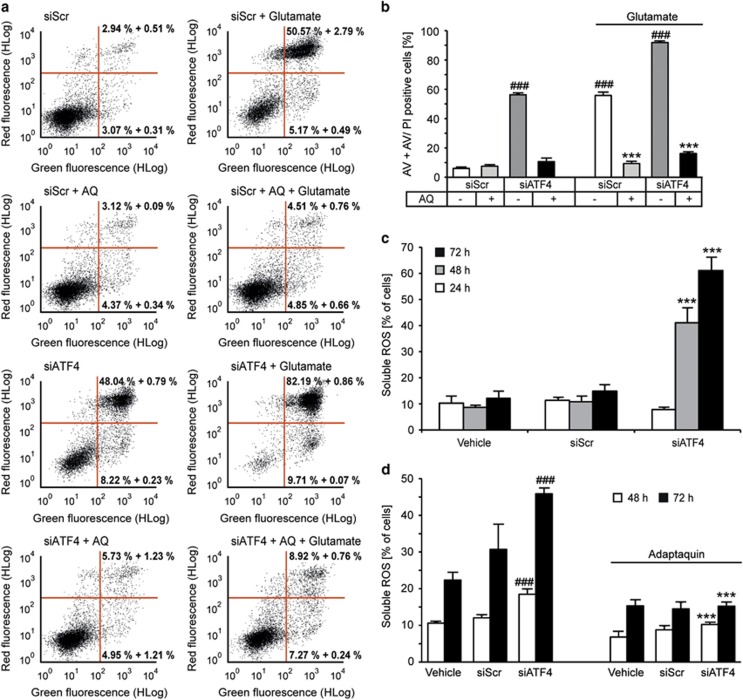
ATF4 silencing does not diminish AQ-mediated protection. (**a**) Representative dot plots show cell death after glutamate challenge (6 mM, 25 h) analyzed by AV/PI staining and subsequent FACS analysis. (**b**) Quantification of AV- and AV/PI-positive cells from the plots in (**a**) (*n*=3). Data are shown as mean±S.D. ^###^*P*<0.001 compared with untreated siScr; ***P<0.001 compared with glutamate-treated siScr (ANOVA, Scheffé's test). (**c**) Quantification of DCF staining reveals time-dependent formation of soluble ROS after ATF4 gene silencing. Data are shown as mean±S.D (*n*=4). ***P<0.001 compared with respective vehicle. (**d**) Cotreatment with AQ prevents generation of soluble ROS after ATF4 knockdown revealed by DCF staining and subsequent FACS analysis. Data are given as mean±S.D. (*n*=3). ^###^*P*<0.001 compared with respective untreated vehicle; ***P<0.001 compared with respective untreated siATF4

To answer the question of whether the restoration of ATF4 expression induced by AQ after 14 h of glutamate challenge was required for the protective effect, we analyzed cell viability after siRNA-mediated gene silencing of ATF4. Surprisingly, AQ still protected ATF4-silenced cells against glutamate-induced cell death. Notably, siRNA-mediated ATF4 downregulation itself resulted in pronounced cytotoxicity, which was completely abolished by AQ (Figures 8a and b). Such toxic effects by ATF4 gene silencing can likely be attributed to the enhanced formation of soluble ROS, which occurred in a time-dependent manner in HT-22 cells after incubation with ATF4 siRNA (Figure 8c). Cotreatment with AQ during transfection prevented this formation of ROS by ATF4 silencing (Figure 8d). Overall, these data suggest that the observed AQ-mediated upregulation of ATF4 was dispensable for the protective effect of the HIF-PHD inhibitor.

## Discussion

The present study clearly depicts that both pharmacological inhibition and gene silencing of HIF-PHDs prevent mitochondrial impairment and cell death in a model of neuronal oxytosis. Our results on cell viability confirm previous findings for PHD1 silencing and the HIF-PHD inhibitors DFO, DHB and CPO in similar models of neuronal oxidative stress.^14, 20, 43^ Protective effects of DFO and dimethyloxalylglycine (DMOG) against glutamate toxicity, and of DFO, DHB and CPO against 3-nitropropionic acid,^11^ were previously also obtained in HIF-1*α*-silenced cells.^14, 44^ Beyond previous findings, our study now demonstrates that HIF-PHD inhibitors exert protective effects at the level of mitochondria. Results from previous studies suggest that HIF-1*α*-independent mechanisms contribute to the protection of mitochondria. The identification of additional targets of HIF-PHDs besides HIF-1*α*, such as the large subunit of RNA polymerase II^16^ and cyclin D1,^17^ and the presence of HIF-PHDs in organisms not expressing HIF isoforms further support the conclusion of HIF-independent functions of HIF-PHDs.^45^ Moreover, HIF-1*α* is predominantly regulated by PHD2.^46^ Nevertheless, in our system we achieved both the protection of cell viability and mitochondrial integrity by silencing PHD1 without regulating the mRNA level of PHD2 (Supplementary Figure 1a), which facilitated an HIF-independent mechanism of protection. Further, Aragonés *et al.*^12^ also showed that PHD1 deficiency reduced the formation of oxidative stress and preserved mitochondrial function after ischemia in skeletal muscle fibers, thereby leading to hypoxia tolerance independently of HIF-induced adaptive effects.

The conservation of mitochondrial function as a crucial mechanism of neuronal protection has widely been shown in AD^47^ and PD^48^ models as well as in humans.^49^ Our present data suggest that mitochondrial protection also has a central role in AQ- and PHD1 CRISPR/Cas9- or siRNA-mediated protection of HT-22 cells. During oxidative stress, mitochondrial demise is induced by the transactivation of the proapoptotic protein BH3-interacting domain death agonist (Bid) via ROS.^9^ In HT-22 cells, these ROS result from lipid peroxidation^5, 30^ due to GSH depletion.^34^ Both silencing PHD1 and AQ abolished the formation of lipid peroxides, indicating an action upstream of mitochondria and leading to mitochondrial protection. Despite the pronounced neuroprotective effect, AQ was unable to prevent the glutamate-induced decrease of GSH levels below detection limits. This observation is in line with previous findings where full protection of HT-22 cells was achieved despite fully depleted GSH levels.^34, 50^ Taken together, these results suggest an influence of HIF-PHD inhibition by AQ at the level of 12/15-LOXs. This can not only be explained by the iron-chelating effects or off-target inhibition of these enzymes, as PHD1 gene silencing also reduced lipid peroxidation (Figure 2a and Supplementary Figure 2a). Further, structural analyses revealed that AQ did not fit to the active center of 12-LOX.^27^ A possible explanation for the activity of HIF-PHD inhibition on LOX activities was recently provided by Karuppagounder and Ratan^51^ who proposed a suppression of 12-LOX expression upon inhibition of HIF-PHDs. This suppression has been attributed to modification of RNA polymerase II.^51^ Downregulation of 12/15-LOX expression after both AQ treatment and PHD1 knockdown further facilitated this assumption (Supplementary Figures 4b–d).

In our system, both PHD1 gene silencing by CRISPR/Cas9 and the use of pharmacological approaches permanently protected HT-22 cells. In contrast, knockdown of PHD1 by siRNA only showed partial protective effects. Apparently, the transient knockdown via siRNA results in a residual activity of the enzyme, which is sufficient enough to promote cell death. Further, inhibition of PHD1 may improve oxygen radical detoxification and accordingly reduced detection of lipoxygenation through shunting glucose into the pentose phosphate pathway, as recently demonstrated in PHD1^−/−^ neurons and in a model of cerebral ischemia *in vivo.*^52^ Further studies should reveal whether such reprogramming of the glucose metabolism is also involved in the protective effects of AQ and other PHD inhibitors in paradigms of oxidative cell death and at the level of mitochondria.

The pharmacological HIF-PHD inhibitors do not act on specific isoforms of the enzyme, suggesting that also inhibition of PHD2 and PHD3 may contribute to the protective effects of the pharmacological HIF-PHD inhibitors against glutamate-induced cell death. However, knockdown of PHD1 by CRISPR/Cas9 was just as effective inferring that PHD2 and PHD3 do not have a significant role during oxytosis. This assumption is in line with previous findings where knockdown of both isoforms did not abolish oxidative-stress-induced death in cortical neurons.^14^ In contrast, overexpression of PHD3 (but not PHD1 or PHD2) induces apoptosis in PC12 cells^53^ and also enhanced the expression of SM-20, a rat orthologue of PHD3 that promotes cell death in PC12 cells^54^ and sympathetic neurons,^55^ indicating a proapoptotic function of PHD3.

PHD1 and PHD3 have been shown to regulate and interact with ATF4,^40, 41^ a transcription factor displaying both antiapoptotic^56, 57^ and proapoptotic properties,^38, 58^ depending on the cellular system and experimental settings. According to observations of Köditz *et al.*^41^ DMOG and siRNA-mediated PHD3 gene silencing stabilized ATF4 expression under normoxic conditions in HeLa cells.^41^ In the present study, AQ-mediated HIF-PHD inhibition restored ATF4 levels after the glutamate challenge. These data, together with the observed toxic effects of ATF4 downregulation, suggest an antiapoptotic function of ATF4 in HT-22 cells. This conclusion is in line with previous findings, where an overexpression of ATF4 protected against glutamate toxicity.^42^ In contrast to the present data, this protection was mediated by an ATF4-dependent upregulation of the xCT via the eIF2*α* pathway and subsequent restoration of GSH levels.^39^ AQ also maintained xCT levels after glutamate exposure. However, this neither correlated with the phosphorylation of eIF2*α* nor restored GSH levels, suggesting that this pathway was not essential to mediate protection by AQ. In this study, AQ even protected ATF4-silenced HT-22 cells from oxytosis and reduced the formation of soluble ROS generated by ATF4 downregulation. This corresponds with earlier results using ATF4^−/−^ cells cultured in the absence of reducing agents where DFO increased the survival, presumably because of the diminished formation of ROS.^57^ All in all, these data imply that, despite potential protective effects of ATF4, the regulation of this transcription factor was dispensable for AQ-mediated protection. Overall, transcriptional regulation by AQ was unlikely the dominating mechanism of action in the present paradigm of oxytosis, as AQ-mediated protection even when applied 10 h after the onset of glutamate exposure.

In conclusion, the present study introduces inhibition of HIF-PHDs as an eligible and promising target to abolish mitochondrial impairment and subsequent neuronal death. The current data suggest that both AQ and selective PHD1 gene silencing act upstream of mitochondria. The mechanism by which AQ prevents glutamate-induced oxidative stress includes multimodal regulations of enzymes involved in the regulation of oxidative stress that are independent of HIF-1*α* regulation.

## Materials and Methods

### Cell culture

HT-22 cells were cultured in Dulbecco's modified Eagle's medium with the addition of 10% heat-inactivated fetal calf serum, 100 U/ml penicillin, 100 mg/ml streptomycin and 2 mM glutamine.

For inducing cell death, 3–7 mM glutamate or 600–800 *μ*M H_2_O_2_ was added to the medium for the indicated amount of time.

SiRNA transfections were performed by using Lipofectamine RNAiMax (Invitrogen, Karlsruhe, Germany) following the manufacturer's protocol. After complex formation, an adequate number of cells was added in antibiotic-free medium to the transfection mix according to the following experimental procedure. Cells were treated after growing for 48 h. The following siRNA sequences were used: 5′-AUCGAAGUCAAACUCUUUCUU-3′ (ATF4), 5′-CAGUGUGGCUGGCGGUCAU-3′ (PHD1 siRNA II), 5′-GCCAUCACUGUCUGGUAUU-3′ (PHD1 siRNA I), 5′-UAAUGUAUUGGAACGCAUA-3′ (scrambled siRNA). AQ, DFO, DHB and CPO were dissolved in DMSO.

### Generation of HT-22 cells with reduced PHD1 protein levels

WT HT-22 cells were seeded in 6-well plates at a density of 100 000 cells/well, and after 24 h, transfected with 5 *μ*g PHD1 CRISPR plasmid (U6gRNA-Cas9-2 A-GFP; MM0000478768, Sigma-Aldrich, Taufkirchen, Germany) using 4.5 *μ*l Attractene and up to 250 *μ*l OptiMEM/well. Two days later, transfection efficiency was confirmed by fluorescence microscopy and cells harvested with trypsin. Afterwards, cells were sorted via FACS and one, two or five cells seeded into a 96-well plate. To exclude dead cells, cells were costained with DAPI. Cells were cultured with weekly media change to obtain appropriate amount of cells for further analysis. Clone 2.17 was derived from initial seeding with two cells per well and clone 5.18 from five cells per well.

### Cell viability

Cytotoxicity and cell viability were quantified by the MTT assay. After induction of cell death by glutamate for indicated time of treatment, MTT was added in a final concentration of 0.5 mg/ml to the culture medium. For calcium chelation experiments, the culture medium with or without extracellular calcium was prepared with indicated EGTA concentrations. In case of H_2_O_2_ treatment, the culture medium was removed after induction of cell death and replaced by PBS containing 0.5 mg/ml MTT. Cells were incubated for 1 h at 37 °C. The resulting purple formazan was dissolved in an appropriate amount of DMSO and absorbance measured at 570 *versus* 630 nm with FluoStar (BMG Labtech, Ortenberg, Germany).

For real-time measurement of cell viability, the xCELLigence system was used as described previously.^59^

Additionally, cell viability after ATF4 gene silencing was detected by an Annexin V/propidium iodide (AV/PI) staining using an Annexin-V-FITC Detection Kit (Promokine, Heidelberg, Germany) followed by FACS analysis. Annexin-V-FITC was excited at 488 nm and emission was detected through a 525/30 bandpass filter. Propidium iodide was excited at 488 nm and fluorescence emission was detected using a 690/50 bandpass filter. Data were collected from 10 000 cells from at least three wells per condition.

### RT-PCR

Forty-eight hours after siRNA transfection, total RNA amount was extracted by InviTrap Spin Universal RNA Kit (Stratec Molecular, Berlin, Germany). RT-PCR was performed with SuperScript III One-Step RT-PCR Kit with Platinum Taq (Invitrogen). The following primers were used: PHD1: forward, 5′-TTGCCTGGGTAGAAGGTCAC-3′ and reverse, 5′-GCTCGATGTTGGCTACCACT-3′ PHD2: forward, 5′-AGCCATGGTTGCTTGTTACC-3′ and reverse, 5′-CTCGCTCATCTGCATCAAAA-3′ GAPDH (glyceraldehyde 3-phosphate dehydrogenase): forward, 5′-AGGCCGGTGCTGAGTAT-3′ and reverse, 5′-TGCCTGCTTCACCACCTTCT-3′. DNA products were visualized on a 1% agarose gel by UV illumination.

### Protein analysis and western blot

Forty-eight hours after siRNA transfection or after the indicated time of glutamate challenge, cells were washed once with PBS and lysed with buffer containing 0.25 M mannitol, 0.05 M Tris, 1 M EDTA, 1 M EGTA, 1 mM DTT, 1% Triton-X, supplemented with Complete Mini Protease Inhibitor Cocktail and PhosSTOP (both Roche Diagnostics, Penzberg, Germany). Extracts were centrifuged at 10 000x*g* for 15 min at 4 °C to eliminate insoluble fragments. The total amount of protein was determined by Pierce BCA Protein Assay Kit (Perbio Science, Bonn, Germany). For western blot analysis, 40–60 *μ*g of protein were loaded on a 12.5% SDS gel and blotted onto a PVDF membrane at 20 mA for 21 h. Incubation with primary antibody was performed overnight at 4 °C. The following primary antibodies were used: anti-PHD1 (Novus Biologicals, Littleton, CO, USA) 1:1 000 in 0.5% IBLOCK, anti-superoxide dismutase 2 (Novus Biologicals) 1:1 000 in 5% skim milk, anti-ATF4 (Santa Cruz Biotechnology, Santa Cruz, CA, USA) 1:500 in 0.5% IBLOCK, anti-xCT (Abcam, Cambridge, UK) 1:1 000 in 5% BSA, anti-eIF2*α* (Cell Signaling, Danvers, MA, USA) 1:1 000 in 5% BSA, anti-phospho-eIF2*α* (Cell Signaling) 1:500 in 5% BSA, anti-15-LOX 1 1:500 in 0.5% IBLOCK (Abcam) and anti-actin C4 (MB Biomedicals, Illkirch, France) 1:10 000 in 5% skim milk. After incubation with proper secondary HRP-labeled antibody (Vector Laboratories, Burlingame, CA, USA), western blot signals were detected by chemiluminescence with Chemidoc software (Bio-Rad, Munich, Germany) and quantified with the Quantity One software (Bio-Rad).

### Mitochondrial ROS formation

Formation of mitochondrial ROS was investigated via MitoSOX red staining (Invitrogen), which shows increasing red fluorescence upon reaction with mitochondrial ROS. For analysis of PHD inhibitors, HT-22 cells were seeded in 24-well plates with 55 000 cells per well. For functional analysis of PHD1 siRNAs, cells were transfected with 22 000 cells per well and grown for 48 h before treatment. At indicated time points after onset of treatment, cells were stained with MitoSOX red for 30 min at 37 °C at a final concentration of 2.5 *μ*M. After collecting and washing once with PBS, cells were resuspended in an appropriate amount of PBS and red fluorescence was detected by FACS analysis. MitoSOX red was excited at 488 nm and emission was recorded using a 690/50 bandpass filter. Data were collected from 10 000 cells from at least three wells per condition.

### Mitochondrial membrane potential

For the analysis of changes in the MMP after glutamate exposure, the MitoPT ΔΨm Kit (Immunochemistry Technologies, Hamburg, Germany) was used. For the analysis of HIF-PHD inhibitors, HT-22 cells were seeded in 24-well plates with 55 000 cells per well. For functional analysis of PHD1 siRNAs, cells were transfected with 22 000 cells per well and grown for 48 h before treatment. After glutamate challenge, in particular, HIF-PHD inhibition cells were collected and stained with TMRE in a final concentration of 200 nM for 20 min at 37 °C. After washing with PBS, cells were resuspended in an appropriate amount of assay buffer and TMRE fluorescence was assessed via FACS analysis. TMRE was excited at 488 nm and emission was recorded using a 690/50 bandpass filter. Data were collected from 10 000 cells from at least three wells per condition.

### Lipid peroxidation

For detection of lipid peroxidation in case of HIF-PHD inhibition, HT-22 cells were seeded in 24-well plates with 55 000 cells per well. To analyze the effects of PHD1 silencing, cells were transfected with PHD1 siRNA with 22 000 cells per well and grown for 48 h. After treatment with glutamate, cells were stained with BODIPY 581/591 C_11_ (Invitrogen) for 1 h at 37 °C in a culture medium at a final concentration of 2 *μ*M. After collecting and washing once with PBS, cells were resuspended in an appropriate amount of PBS. Lipid peroxidation was analyzed by detection of fluorescence shift from green to red via FACS analysis. Excitation was performed at 488 nm and emission was recorded with a 525/30 bandpass filter (green) and a 690/50 bandpass filter (red). Data were collected from 10 000 cells from at least three wells per condition.

### Intracellular soluble ROS

For the analysis of intracellular soluble ROS, cells were seeded in 24-well plates with 55 000 cells per well and treated with glutamate and AQ or transfected with ATF4 siRNA for the indicated amount of time. To analyze the effects of AQ on the formation of soluble ROS in ATF4-silenced cells, AQ was added immediately after transfection to the culture medium. For detection of intracellular soluble ROS, the culture medium was replaced by a medium without serum containing CM-H_2_DCFDA in a final concentration of 2.5 *μ*M. Cells were incubated at 37 °C for 30 min followed by 30 min incubation without dye in serum containing fresh medium. Subsequently, cells were harvested and washed once with PBS. For detection of green fluorescence, cells were resuspended in a suitable amount of PBS and analyzed by FACS. DCF was excited at 488 nm and emission was recorded with a 525/30 bandpass filter. Data were collected from 10 000 cells from at least three wells per condition.

### ATP measurements

For analysis of total ATP levels, cells were seeded in white 96-well plates (8 000 cells per well) and treated with HIF-PHD inhibitors or transfected with PHD1 siRNA (3500 cells per well) and grown for 48 h. At indicated time points of glutamate exposure, ATP levels were analyzed by luminescence detection with FluoStar according to the manufacturer's protocol using the ViaLight plus Kit (Lonza, Verviers, Belgium).

### Measurement of cellular OCR

To determine the cellular OCR, cells were plated in XF96-well microplates (8000 cells per well; Seahorse Bioscience, Copenhagen, Denmark) and treated with HIF-PHD inhibitors and glutamate. After appropriate time, OCR measurements were performed as described previously^60^ with minor modifications. Briefly, the growth medium was washed off and replaced by ~180 *μ*l of assay medium (with 4.5 g/l glucose as the sugar source, 2 mM glutamine, 1 mM pyruvate, pH 7.35) and cells were incubated at 37 °C for 60 min. Three baseline measurements were recorded before adding the compounds. Oligomycin was injected in port A (20 *μ*l) at a final concentration of 3 *μ*M, FCCP (22.5 *μ*l in port B) at a concentration of 0.4 *μ*M and rotenone/antimycin A (25 *μ*l in port C) at a concentration of 1 *μ*M. Three measurements were performed after the addition of each compound (4 min mixing followed by 3 min measuring).

### GSH measurement

To determine GSH levels, HT-22 cells were seeded in 6-well plates (180 000 cells per well). After treatment with glutamate and AQ for indicated amount of time, two wells per condition were harvested by scratching and washed once with PBS. GSH measurements were performed using GSH Assay Kit (Cayman Chemical Company, Ann Arbor, MI, USA) following the manufacturer's protocol. Briefly, cells were resuspended in MES buffer (0.4 M 2-(*N*-mopholino)ethanesulfonic acid, 0.1 M phosphate, 2 mM EDTA, pH 6.0) and homogenized by sonification. Insoluble fragments were removed by centrifugation at 10 000x*g* for 15 min. The supernatant was deproteinated by the addition of an equal volume of metaphosphoric acid (1.25 M). After incubation for 5 min, the mixture was centrifuged at 17 000x*g* for 10 min. Subsequently, the supernatant was mixed with a 4 M solution of triethanolamine to increase the pH. After transferring into a 96-well plate, the assay cocktail containing provided MES buffer, cofactor mixture, enzyme mixture and Ellman's reagent was added. Absorbance was measured at 405 nm after 30 min of incubation. Total GSH amount was determined via standard curve calculation and normalized to protein content.

### Statistical analysis

All data are given as mean±S.D. Statistical comparison between treatment groups was performed by analysis of variance (ANOVA) followed by Scheffé's *post hoc* test. Calculations were executed with Winstat standard statistical software (R Fitch Software, Bad Krozingen, Germany).

## Abbreviations

AD: Alzheimer's disease
AIF: apoptosis-inducing factor
ANOVA: analysis of variance
ATF4: activating transcription factor 4
Bid: BH3-interacting domain death agonist
CPO: Ciclopirox
Cytc: cytochrome *c*
DCF: 5,6-chloromethyl-2′,7′-dichlorodihydrofluorescein diacetate acetyl ester
DFO: deferoxamine
DHB: ethyl-3,4-dihydroxybenzoate
DMOG: dimethyloxalylglycine
eIF2*α*: eukaryotic initiation factor 2*α*
FACS: fluorescence-activated cell sorting
GAPDH: glyceraldehyde 3-phosphate dehydrogenase
GSH: glutathione
HIF: hypoxia-inducible factor
HIF-PHD: HIF-prolyl-4-hydroxylase
LOX: lipoxygenase
MMP: mitochondrial membrane potential
MnSOD: manganese superoxide dismutase
MTT: 3-(4,5-dimethylthiazol-2-yl)-2,5-diphenyltetrazolium bromide
OCR: oxygen consumption rate
PD: Parkinson's disease
ROS: reactive oxygen species
TMRE: tetramethylrhodamine ethyl ester
