# Supplementary material for: Inhibition of HIF-prolyl-4-hydroxylases prevents mitochondrial impairment and cell death in a model of neuronal oxytosis
Source: Cell Death Dis. 2016 May 5;7(5):e2214–. doi: 10.1038/cddis.2016.107 (PMC4917646; doi:10.1038/cddis.2016.107)
Supplement: Supplementary Figures [file cddis2016107x1.pdf]

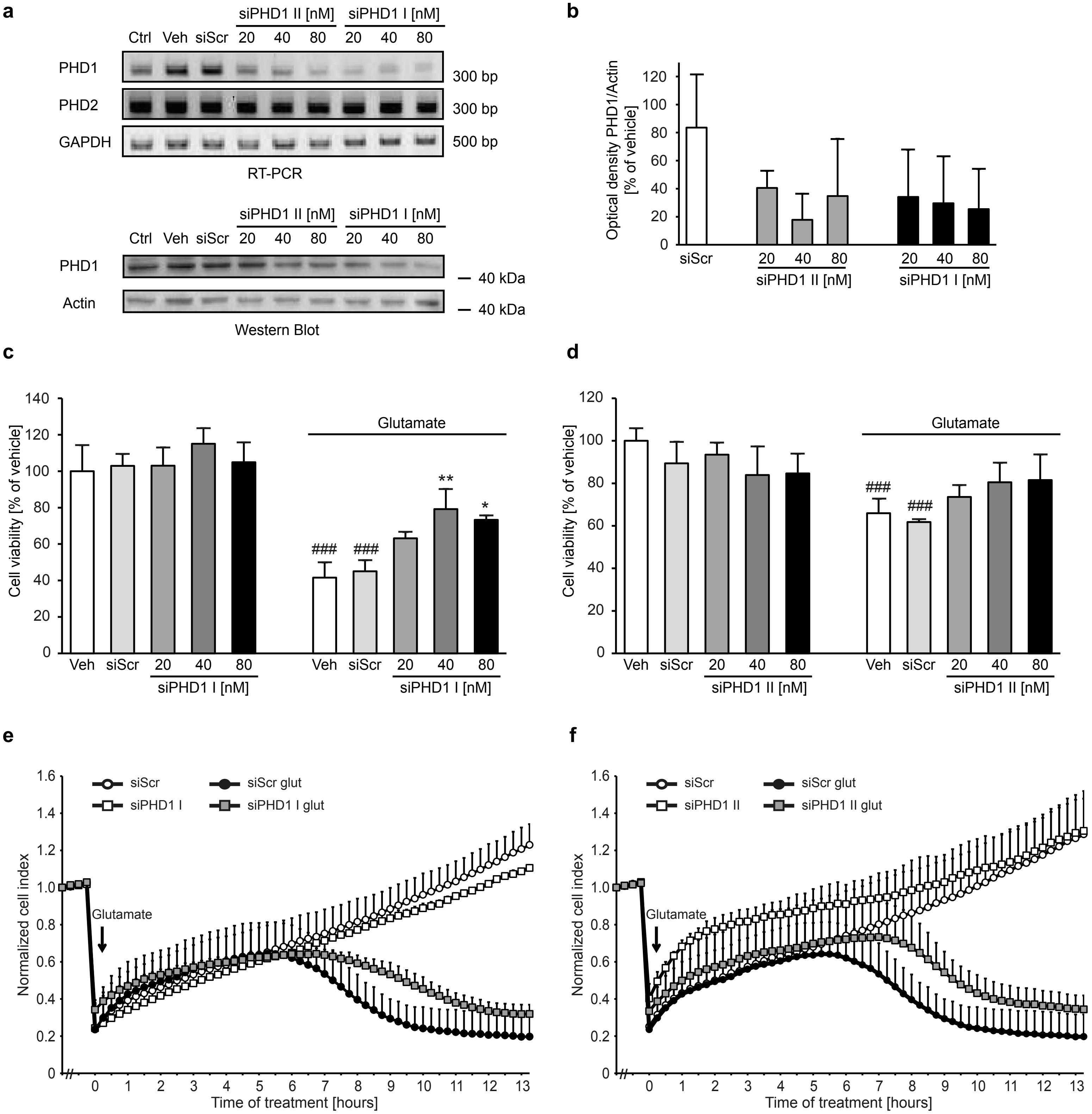

Supplemental figure 1: PHD1 siRNA-mediated gene silencing attenuates glutamate-induced cell death.
a: Selective knockdown of PHD1 by siRNA sequence I and II was verified by RT-PCR and Western blot analysis. **b:** Quantification of 5 independent Western blots shows a reduction of PHD1 protein level. **c, d:** MTT assay revealed small protection of different concentrations of siPHD1 I (**c**) and siPHD1 II (**d**) against glutamate toxicity (5 mM, 14 h) compared to cells transfected with unspecific control siRNA (siScr). Data are given as mean + SD. (n = 4) $^{###}p < 0.001$ compared to untreated vehicle; $^{*}p < 0.05$ and $^{**}p < 0.01$ compared to glutamate-treated vehicle. (ANOVA, Scheffé‘s test). **e, f:** xCELLigence real-time measurement: Cells transfected with siPHD1 I (**e**) or siPHD1 II (**f**) show transient protection against treatment with 5 mM glutamate (glut) compared to cells transfected with siScr.

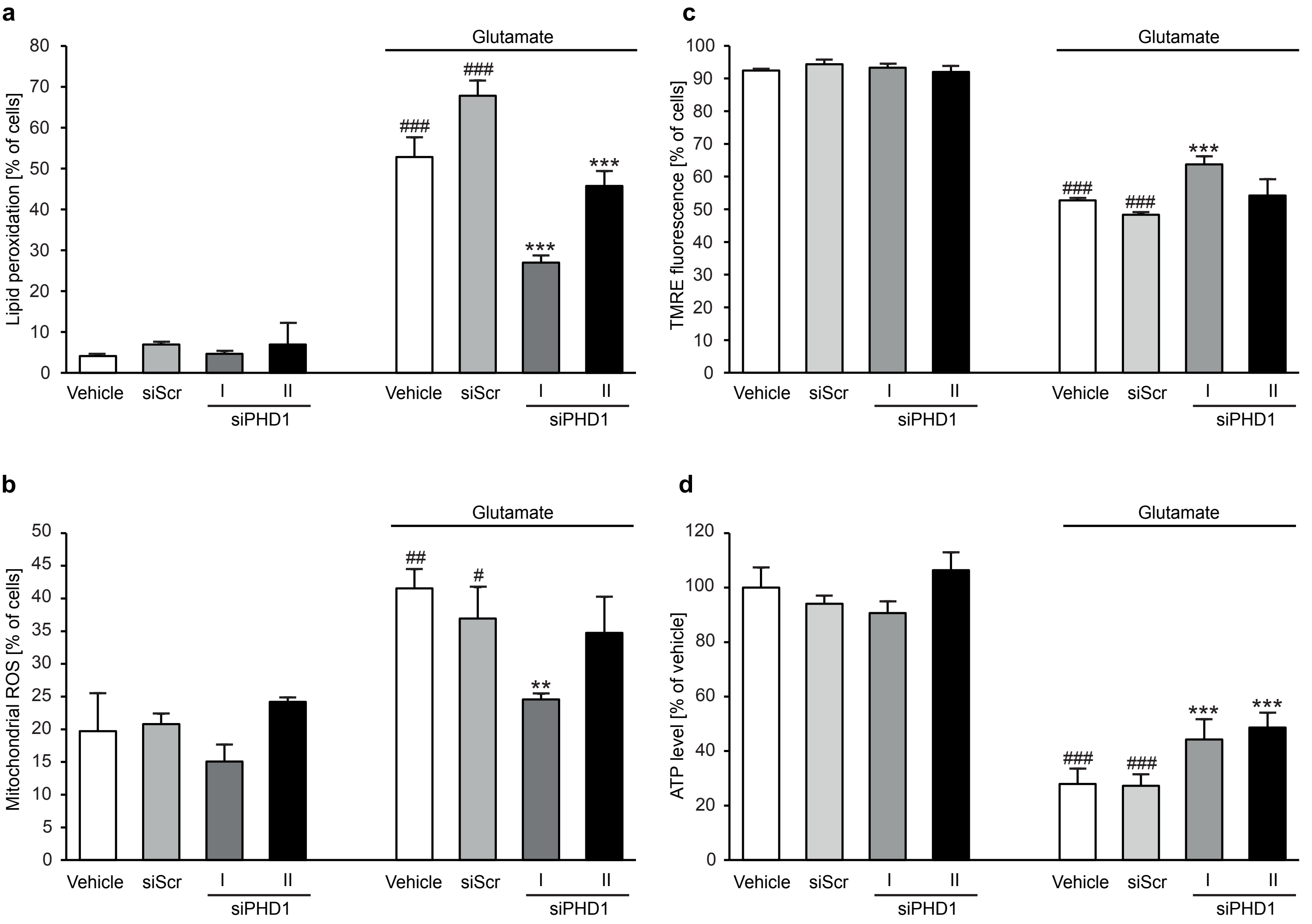

Supplemental figure 2: PHD1 siRNA I and II restore mitochondrial integrity.

a: PHD1 siRNA (siPHD1) I and II significantly reduced glutamate-induced (5 mM, 15 h) lipid peroxide production measured via BODIPY 581/891 staining and subsequent FACS analysis (n=4). Data are shown as mean + SD. $^{###}p < 0.001$ compared to untreated vehicle; $^{***}p < 0.001$ compared to glutamate-treated vehicle. (ANOVA, Scheffé's test). **b:** Mitochondrial ROS production was detected by MitoSOX staining and following FACS analysis. Glutamate treatment led to an increase in ROS production. SiPHD1 I and II reduced this increase. Quantification of MitoSOX fluorescence of n = 3 independent experiments. Data are shown as mean + SD. $^{\#}p < 0.05$ and $^{##}p < 0.01$ compared to untreated vehicle; $^{**}p < 0.01$ compared to glutamate-treated vehicle (ANOVA, Scheffé's test). **c:** SiPHD1 I and II prevented glutamate-induced (5 mM, 11 h) mitochondrial depolarization shown by restored TMRE fluorescence. Data are given as mean + SD (n = 3). ($^{###}p < 0.001$ compared to untreated vehicle; $^{***}p < 0.001$ compared to glutamate-treated vehicle; ANOVA, Scheffé's test). **d:** After 15 h of treatment with glutamate (4 mM) ATP levels were measured. PHD1 silencing prevented glutamate-induced ATP depletion (n = 8). Data are shown as mean + SD. $^{###}p < 0.001$ compared to untreated vehicle; $^{***}p < 0.001$ compared to glutamate-treated vehicle. (ANOVA, Scheffé's test).

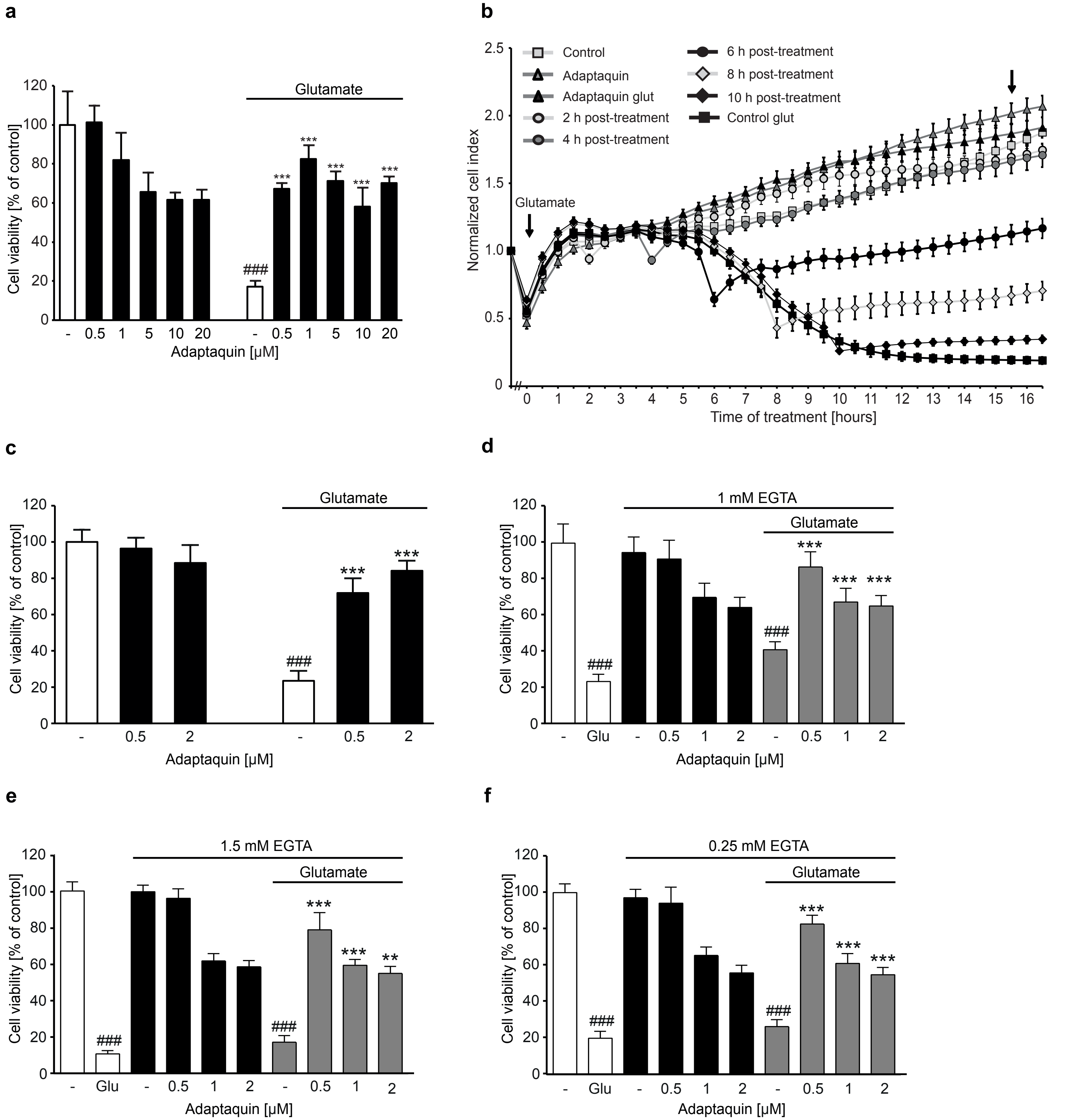

Supplemental figure 3: Adaptaquin prevents glutamate-induced cell death in HT-22 cells.
a: MTT assay depicts protective effect of adaptaquin following glutamate exposure (7 mM, 16 h). **b:** Impedance was measured in post-treatment conditions. Adaptaquin (2 µM) was added 2, 4, 6, 8 and 10 h after the onset of glutamate (4 mM). Adaptaquin reduced loss of impedance compared to glutamate-treated con-trols when applied up to 8 h after the onset of glutamate (n = 7). **c-f:** HT-22 cells were cultured for 24 h in DMEM. Afterwards, cells were treated for 15.5 h with (**c**) 5 mM glutamate in calcium free medium, 6 mM glutamate in culture medium supplemented with (**d**) 1 mM EGTA or (**e**) 1.5 mM EGTA, or (**f**) calcium free medium supplemented with 0.25 mM EGTA. MTT assay revealed that co-treatment with AQ prevented glutamate-induced cell death. Data in **a** and **c-f** are shown as mean + SD. (n = 8) $^{\#\#\#}p < 0.001$ compared to untreated control; $^{**}p < 0.01$ or $^{***}p < 0.001$ compared to glutamate-treated control. (ANOVA, Scheffé's test).

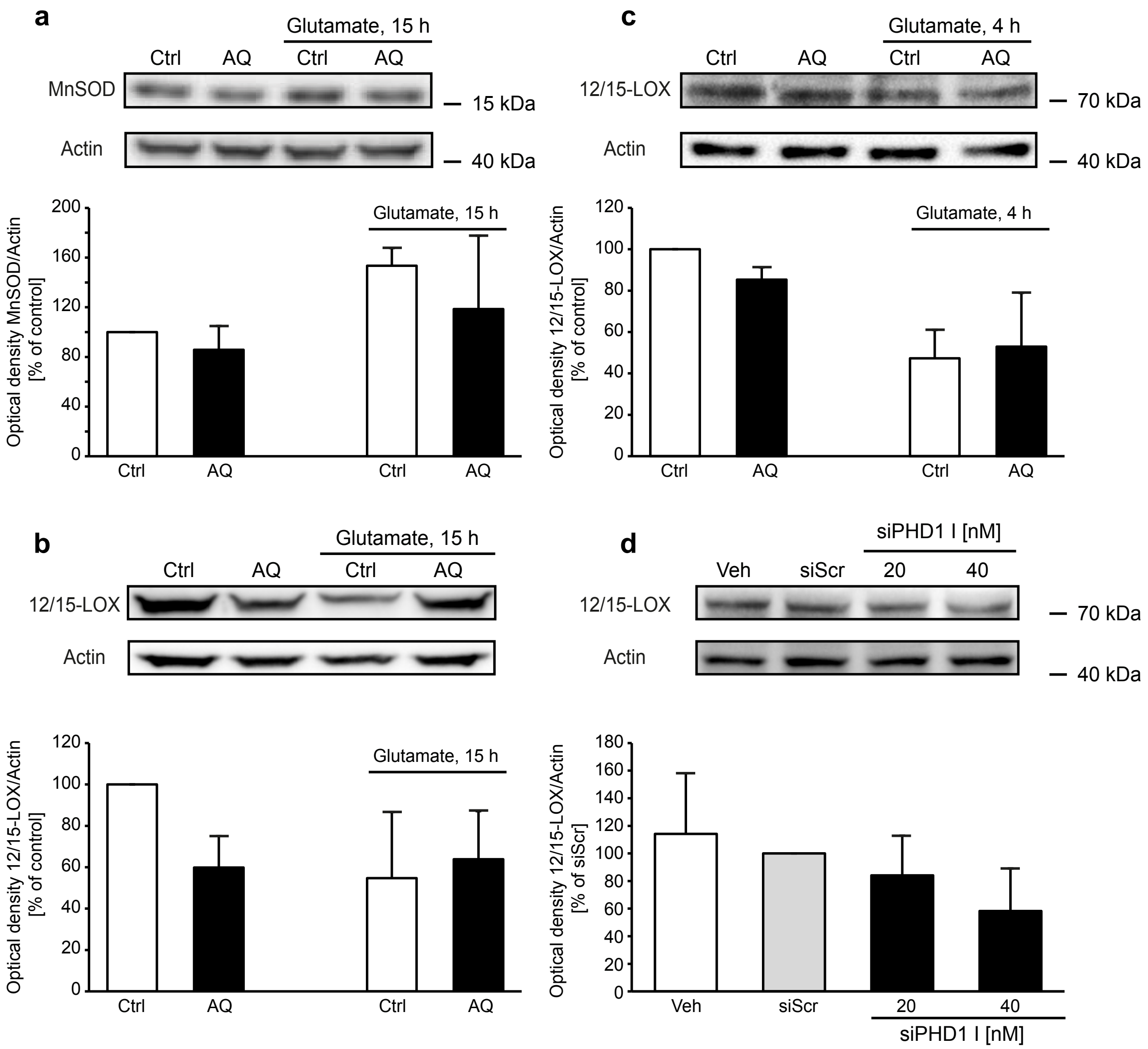

Supplemental figure 4: Adaptaquin alters expression of 12/15-LOX.

a: Representative Western blot and corresponding quantifications of 4 independent experiments show enhanced expression of MnSOD after glutamate (4 mM, 14 h) challenge. **b, c:** Representative Western blot and corresponding quantifications of 4 or 3 independent experiments respectively show a decrease of 12/15-LOX protein levels after treatment with adaptaquin in the presence and absence of glutamate after 4 and 15 h of exposure. **d:** Representative Western blot and corresponding quantifications of 3 independent experiments show reduced expression of 12/15-LOX after knockdown of PHD1 with siRNA sequence I.
